# Habitat Associations of Juvenile Fish at Ningaloo Reef, Western Australia: The Importance of Coral and Algae

**DOI:** 10.1371/journal.pone.0015185

**Published:** 2010-12-07

**Authors:** Shaun K. Wilson, Martial Depczynski, Rebecca Fisher, Thomas H. Holmes, Rebecca A. O'Leary, Paul Tinkler

**Affiliations:** 1 Science Division, Department of Environment and Conservation, Marine Science Program, Kensington, Western Australia, Australia; 2 Australian Institute of Marine Science, The Oceans Institute, University of Western Australia, Crawley, Western Australia, Australia; University of Glamorgan, United Kingdom

## Abstract

Habitat specificity plays a pivotal role in forming community patterns in coral reef fishes, yet considerable uncertainty remains as to the extent of this selectivity, particularly among newly settled recruits. Here we quantified habitat specificity of juvenile coral reef fish at three ecological levels; algal meadows vs. coral reefs, live vs. dead coral and among different coral morphologies. In total, 6979 individuals from 11 families and 56 species were censused along Ningaloo Reef, Western Australia. Juvenile fishes exhibited divergence in habitat use and specialization among species and at all study scales. Despite the close proximity of coral reef and algal meadows (10's of metres) 25 species were unique to coral reef habitats, and seven to algal meadows. Of the seven unique to algal meadows, several species are known to occupy coral reef habitat as adults, suggesting possible ontogenetic shifts in habitat use. Selectivity between live and dead coral was found to be species-specific. In particular, juvenile scarids were found predominantly on the skeletons of dead coral whereas many damsel and butterfly fishes were closely associated with live coral habitat. Among the coral dependent species, coral morphology played a key role in juvenile distribution. Corymbose corals supported a disproportionate number of coral species and individuals relative to their availability, whereas less complex shapes (i.e. massive & encrusting) were rarely used by juvenile fish. Habitat specialisation by juvenile species of ecological and fisheries importance, for a variety of habitat types, argues strongly for the careful conservation and management of multiple habitat types within marine parks, and indicates that the current emphasis on planning conservation using representative habitat areas is warranted. Furthermore, the close association of many juvenile fish with corals susceptible to climate change related disturbances suggests that identifying and protecting reefs resilient to this should be a conservation priority.

## Introduction

Settlement of fish from the pelagic environment and the subsequent recruitment into benthic populations are key processes in determining the structure of fish communities. Whilst considerable debate has centered on the relative importance of density dependant and independent factors in structuring adult communities [1], [2], [3], it seems the availability of suitable habitat plays a key role for most species at some spatial scale [4], [5], [6]. At larger scales, many species of fish recruit to particular ecosystems, such as seagrass meadows, mangrove habitats or coral reefs [7], [8]. On a smaller scale, juveniles of some species are known to reside within very specific microhabitats, such as particular coral species [9]. Understanding the reliance of fish on specific habitats and the spatial scale at which different habitats are important is crucial for developing appropriate conservation strategies and successful management of fish communities.

Coral reef ecosystems provide habitat for thousands of species [10], [11], however their level of dependence on reef habitat varies greatly among species, can change with spatial scale and may differ among life stages. Many species recruit and settle directly on the reef itself, or specific micro-habitats within the coral reef [12], [13]. However, some species recruit to seagrass or mangroves adjacent to coral reefs, migrating to coral reefs only as sub-adults [7], [8], [14], [15]. Moreover, some species may live on coral reefs but have no obvious reliance on the living corals that provide much of the habitat. Indeed, adult fish that feed exclusively on coral or shelter among the branches of live coral colonies represent only 10% of species on reefs [16], [17]. While the adults of many species may not be directly associated with live coral, some species may settle as larvae in live coral and spend much of their early life history associated with them. This dependence on live coral habitat as juveniles could explain why a decline in coral cover is often reflected by a reduction in fish species with no apparent coral affinity as adults [16], [18]. Furthermore, the structural complexity provided by coral skeletons provides a predator refuge for both adult and juvenile fish, even after corals die. Studies have shown loss of small bodied fishes associated with the collapse of coral skeletons [19], [20], whereas species richness remains stable when structural complexity is retained [21], [22].

A decline in coral cover at regional and local scales [23], [24], [25] raises concerns about the effects this may have on fish that recruit to live coral habitat and the consequences for reef fish communities as a whole [18]. To date most studies on the importance of coral as habitat for juvenile reef fish have been based on experiments and field observations in the Pacific Ocean [26], [27], [28]. These studies have tended to focus on coral-associated species from prominent families such as, pomacentrids [27], [28], [29], chaetodontids [30] or gobies [9], on reefs within the Great Barrier Reef, Australia. However, to better understand the importance of coral to juvenile reef fish and the implications of coral decline, research needs to assess habitat specialization across a broader suite of species, considering different reef environments and locations [31]. Moreover, consideration must be given to the importance of habitats in close proximity to coral reefs which may also be under threat. Previous studies have examined the significance of seagrass and mangroves as nursery grounds for fish [7], [8], [32], [33], but other habitats may also be important. For example, rubble areas adjacent to reefs are the preferred habitat of some Caribbean juvenile fish [12].

Here we look at habitat associations of juvenile fish along 300km of Ningaloo Reef located on the eastern margin of the Indian Ocean. We examine habitat preferences of both ecological and fisheries important juvenile fish, determining if each species is associated with live coral, coral skeletons and different coral growth forms. We also assess the probability of observing juvenile fish in algal meadows, which are a prominent feature of coastal systems along the tropical coast of Western Australia, often occurring in close proximity to coral reefs [34]. This information will; improve our ability to predict effects of coral loss on reef fish assemblages, identify the potential importance of nursery habitats and advance our capacity to manage reef fish communities.

## Methods

Habitat use by juvenile fish was assessed using underwater visual surveys at 9 locations in 2009 and 21 in 2010. Surveys were carried out at coral sites located on the back-reef area, (5 in 2009, 13 in 2010) and within algal meadows in the lagoon (4 in 2009, 8 in 2010). Coral sites typically had 10–80% live coral cover (38±2% mean, standard error) and algal meadows were characterized by high coverage of phaeophytes (46±2%), such as *Sargassum* spp. and *Dictyota* spp. and <5% living coral or coral skeletons coverage. All surveys were carried out in 1 to 4 m water depth. At each location 3 to 9 transects, 30×1 m, were surveyed for juvenile fish. Overall there were 135 transects carried out on coral reefs and 79 on algal meadows. Within each transect, juvenile fish were identified to species and the microhabitat immediately beneath them when first observed was recorded. Categories used to describe microhabitats were: live coral, dead coral, fleshy macroalgae, rubble and sand. The live coral microhabitat was further categorized as: branching, corymbose, encrusting, foliaceous, massive, plate or submassive, based on the growth forms described in [35]. At the completion of each transect, observer's also estimated percent benthic cover of the aforementioned microhabitats. This technique provides a quick and reliable estimate of microhabitat availability; similar to estimates obtained using line intercept transects [36]. We also recorded if juveniles were in groups and counted the number of fish within the group, as coral reef fish often recruit to habitats occupied by conspecifics and this may influence their choice of microhabitat [37], [38], [39]. All observers had been trained in fish and habitat identification prior to data collection and a pilot study indicated there was no significant difference between observer's ability to identify and record juvenile fish abundance [40]. Fish were identified as juveniles based on colouration and body size (<4 cm total length, and <25% of maximum adult total length).

Juvenile habitat preferences were assessed at several ecological scales. At the largest scale (10 s metres) we examined the probability of species being found in coral dominated backreefs and lagoon algal meadows. At an intermediate scale (metres) we examined the use of live and dead coral microhabitats as well as preferential use of different growth forms of live coral. Use of coral or algal sites and live or dead coral was assessed for all species where 5 or more juvenile fish were observed during the course of the study. Use of different coral growth forms were however only assessed among those species shown to have preference for live coral and observed on 12 or more transects. These criteria allowed a reasonable measure of variation among habitats when assessing species choice between habitat categories.

Bayesian logistic regression was used to determine the probability of each species being found on the coral dominated backreef habitat and the lagoon algal meadows [41], [42]. The observed presence/absence data for each species were modeled as *y_i_* ∼ Bernoulli(*p_i_*), where *y_i_* is the presence (1) or absence (0) of a species on each transect for *i* = 1…*N* obervations. Then logit(*p_i_*)  =  *α_0_ + α_1_ × x_i_ + λ_li_*, where *x* is a indicator or dummy variable denoting a transect as being either from a coral dominated backreef habitat or an algal meadow. The coefficients *α_0_* and *α_1_* were modeled as normal distribution, *α* ∼ Normal (0, *σ^2^*), where *σ^2^* was set to a large constant, and thus both coefficients were treated as non-informative priors. The random effect of location (*l = *1, …,16) was incorporated via the variable *λ,* and modeled as *λ_l_ ∼ Normal*(*0,σ^2^*). Bayesian logistic regression was performed using Markov chain Monte Carlo (MCMC) using the library BRugs, an R (http://cran.r-project.org/) interface to OpenBUGS (http://www.openbugs.info/w/). Convergence of models was assessed using all convergence diagnostics in the CODA package [43], in particular cross-correlations and accuracy of estimating quantiles [44]. From these diagnostics the burn-in selected was 1,000 iterations and a further 10,000 iterations were used to estimate the parameters α*_0_* and α*_1_*.

To assess if juvenile fish preferentially associate with live coral, dead coral or coral structure more generally (live and dead coral as a combined habitat) we examined all transects where a species occurred and calculated the percentage of groups occurring in live or dead corals, along with the sum of these percentages. Mean values with 95% confidence intervals were used to assess if species used these two habitats disproportionately relative to their availability. For example, if the lower bound of the 95% confidence limit around the mean percentage of fish in live coral microhabitats did not intercept estimates of mean live coral cover, it was deemed that species preferentially associated with live coral. Similar calculations were used to assess preferential use of coral skeletons (dead coral) and the physical structure provided by both live and dead corals.

Preferential use of coral growth forms was assessed among 10 species of coral associated species using selectivity indices. For each fish species indices were calculated as the proportion of fish groups that used a coral growth form, divided by the proportion of benthos occupied by that growth form [45]. Relevance of indices was interpreted using 95% confidence intervals [46]. When the lower bounds of the 95% confidence interval were >1, this suggested preferential use of a coral growth form.

## Results

A total of 6979 juvenile fish representing 11 families and 56 species were observed during the study (Table 1). Some of these species aggregated on the same microhabitat, often forming large groups of individuals. For example, there were 447 individuals of *Chromis viridis* observed during surveys, but only 22 groups and there was usually only one or two groups within a single transect. Other species were less gregarious, the number of individuals observed being similar to the number of groups (e.g. *Chaetodon trifascialis*).

**Table 1:** Juvenile fish observed on coral and algal reefs, Ningaloo.

Family	Species	Individuals	Groups	Transects
Acanthuridae	*Acanthurus grammoptilus*	40	26	18
Acanthuridae	*Acanthurus dussumieri*	12	7	4
Apogonidae	*Apogon rueppellii* a	128	18	8
Apogonidae	*Apogon wassinki*	332	22	20
Apogonidae	*Cheilodipterus quinquelineatus*	20	18	11
Blenniidae	*Atrosalarias fuscus*	13	10	8
Chaetodontidae	*Chaetodon assarius*	24	21	17
Chaetodontidae	*Chaetodon auriga*	6	6	5
Chaetodontidae	*Chaetodon plebeius* *	116	95	55
Chaetodontidae	*Chaetodon trifascialis* *	14	14	12
Chaetodontidae	*Chaetodon trifasciatus* *	7	6	5
Labridae	*Anampses geographicus*	234	42	27
Labridae	*Anampses caeruleopunctatus*	11	9	9
Labridae	*Coris aygula*	6	4	4
Labridae	*Coris caudimacula*	158	66	37
Labridae	*Cheilinus chlorourus*	17	14	8
Labridae	*Cheilinus trilobatus*	59	56	33
Labridae	*Cheilio inermis*	20	10	8
Labridae	*Gomphosus varius*	12	12	11
Labridae	*Hologymnosus annulatus*	9	8	7
Labridae	*Halichoeres marginatus*	11	11	8
Labridae	*Halichoeres nebulosus* a	203	132	38
Labridae	*Hemigymnus fasciatus*	8	7	7
Labridae	*Hemigymnus melapterus*	33	32	23
Labridae	*Labrichthys unilineatus* *	155	97	51
Labridae	*Labroides dimidiatus*	18	16	13
Labridae	*Macropharyngodon ornatus*	35	33	23
Labridae	*Stethojulis bandanensis*	442	314	106
Labridae	*Stethojulis interrupta*	431	230	79
Labridae	*Stethojulis strigiventer*	219	92	42
Labridae	*Thalassoma hardwicke*	33	31	20
Labridae	*Thalassoma lunare*	354	243	95
Labridae	*Thalassoma lutescens*	26	25	16
Lethrinidae	*Lethrinus atkinsoni*	105	66	22
Monocanthidae	*Oxymonacanthus longirostris* *	7	4	4
Mullidae	*Parupeneus barberinoides*	47	28	18
Mullidae	*Parupeneus spilurus*	149	49	32
Nemipteridae	*Scolopsis bilineatus*	7	7	6
Pomacentridae	*Chromis viridis* *	447	29	22
Pomacentridae	*Dascyllus aruanus* *	213	101	52
Pomacentridae	*Dascyllus reticulatus* *	109	47	30
Pomacentridae	*Dascyllus trimaculatus* *	21	8	8
Pomacentridae	*Dischistodus perspicillatus*	5	4	4
Pomacentridae	*Dischistodus prosopotaenia*	8	8	4
Pomacentridae	*Neoglyphidodon melas*	26	20	14
Pomacentridae	*Pomacentrus coelestis*	748	182	69
Pomacentridae	*Pomacentrus moluccensis* *	632	324	94
Pomacentridae	*Pomacentrus vaiuli*	60	59	29
Pomacentridae	*Plectroglyphidodon lacrymatus*	55	51	37
Pomacentridae	*Stegastes nigricans*	10	8	6
Pomacentridae	*Stegastes obreptus*	10	10	9
Scaridae	*Chlorurus microrhinos*	26	22	18
Scaridae	*Chlorurus sordidus*	516	226	85
Scaridae	*Leptoscarus vaigiensis* a	410	91	40
Scaridae	*Scarus frenatus*	71	49	35
Scaridae	*Scarus prasiognathos*	91	68	44

Data is only presented for species where 5 or more individuals where observed. “Individuals” represents the total number of fish and “Groups” the number of aggregations seen for that species. “Transects” represents the number of transects on which that species was observed. Total number of transects, 214.

*Species known to be closely associated with live coral as adults [18].

a Species where adults are predominantly found in algal habitats. All other species were commonly observed on coral reefs as adults.

**Figure 1:**
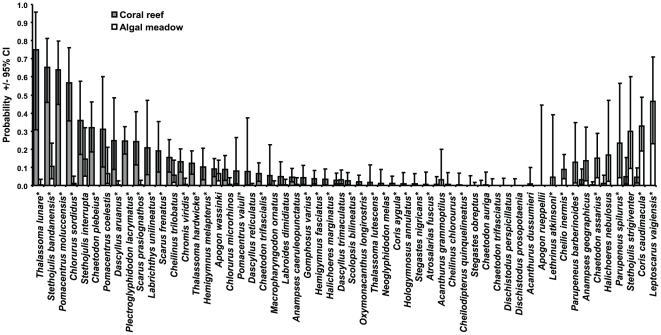
Occurrence of juvenile fish on coral reefs and algal meadows at Ningaloo. Probabilities of observing fish in each habitat based on presence of species on 135 coral reef and 79 algal meadow transects. * Species where credible intervals (CI) for probability of occurrence in algal and coral reef habitats do not overlap.

Most juvenile species (49, 88%) were seen at least once on coral reefs and 25 (45%) species were only ever observed within this habitat (Figure 1). Fewer species were observed on algal sites (31, 55%), although there were 7 (13%) species unique to this habitat. Four of these species (*Lethrinus atkinsoni*, *Cheilio inermis, Parupeneus spilurus and P. barberinoides*) have been frequently observed on coral reefs (Table 1), suggesting ontogentic shifts in habitat use. The scarid, *Leptoscarus vaigiensis,* was also common and unique to algal meadows, although adults were not observed on coral reefs (Table 1). Juveniles of 24 species occurred at both coral and algal sites, although the probability of observing these within each habitat varied (Figure 1). In particular, the probability of observing the pomacentrids *Chromis viridis* and *Pomacentrus moluccensis* was significantly greater on coral reefs. Conversely, the probability of finding juveniles of the labrid *Coris caudimacula* and the butterflyfish, *Chaetodon assarius,* was greater on algal meadows than coral reefs.

**Figure 2:**
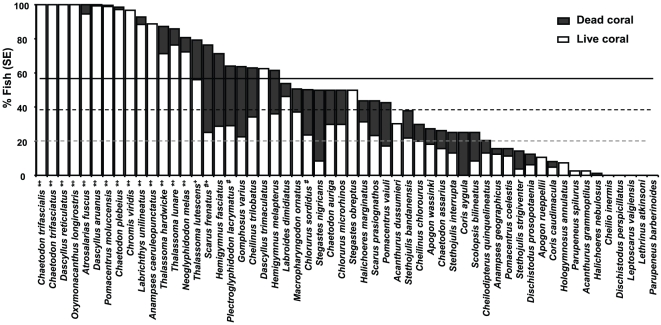
Occurrence of juvenile fish on live and dead coral habitats. Mean percentages calculated from percent of fish groups observed on either live or dead coral on each transect for which that species was observed. * preferential use of live coral, based on lower bounds of 95% confidence interval for live coral use being greater than mean live coral cover (38%, middle dash line). # preferential use of dead coral, based on lower bounds of 95% confidence interval for dead coral use being greater than mean dead coral cover (20%, lower gray dash line). + preferential use of coral (live + dead), based on lower bounds of 95% confidence for use being greater than the mean cover of live and dead coral cover combined (58%, upper solid line).

Approximately 38% of all juvenile fish were closely associated with live coral microhabitats, although some species had a greater affinity for live coral than others (Figure 2). The percentage of fish observed on live coral was significantly greater than the mean live coral cover on reefs (38±2%) for 14 species, suggesting a preference for live coral microhabitats (Figure 2). The majority of these were fish species that feed or closely associate with live coral as adults (Table 1). However three species of labrid (*Thalassoma hardwicke, T. lunare, Anampses caeruleopunctatus*) are not known to feed or closely associate with live coral as adults (Table 1). Three species of juvenile fish (*Chlorurus sordidus, Plectroglyphidodon lacrymatus, Scarus frenatus*) were also closely associated with coral skeletons, with a higher percentage occurring among the skeletons of dead corals than the average cover of dead corals on reefs (Figure 2, 20±2%).

**Figure 3:**
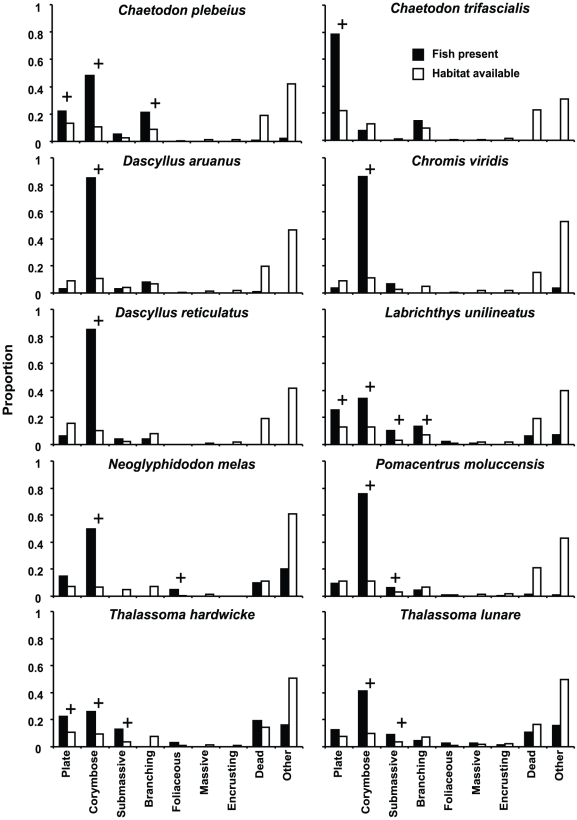
Distribution of 10 juvenile fish species among coral growth forms. Proportion of fish in each habitat calculated from number of “groups” of that species observed in that microhabitat. Habitat availability for each fish species calculated only from transects where that species observed. + preference for microhabitat, based on lower bound of 95% confidence limits about selectivity indices >1.

For juveniles showing a strong live coral affinity, the actual coral growth forms used varied amongst species (Figure 3). Nine of ten species were found in close association with corymbose corals. The five pomacentrid species particularly preferred corymbose corals, more than half of the fish seen for each species being sighted in this microhabitat. The proportion of labrids seen on corymbose corals was also high, especially when fish were newly settled and very small. However, the three labrid species used a broader suit of coral growth forms than the pomacentrids, showing preferences for plate, submassive and branching corals, in addition to corymbose growth forms. The butterflyfish, *Cheatodon trifascialis*, was the only species with low occurrence on corymbose corals, juveniles of this species being predominantly found in plate corals. Other coral growth forms were rarely used, less than 1% of juveniles affiliating with massive or encrusting corals and no indication of preferential use of these coral types by any fish species.

## Discussion

The composition of juvenile fish communities on coral reefs and macroalgal meadows are distinctly different, with the majority of fish species observed predominantly within one of these habitats. This implies that both coral and macroalgal areas can be considered essential juvenile habitat and that high fish diversity is likely to be dependant on the presence of both habitats. Moreover adults of juveniles observed exclusively in algal meadows were frequently observed on coral reefs, suggesting some connectivity between the two habitats. This process is analogous to the ontogenetic habitat shift observed between seagrass meadows and coral reefs [7], [8], [15] and emphasizes the importance of the presence of algal beds adjacent to coral reefs for some fish species.

Juvenile fish of ecological or fisheries importance were also observed in either coral reefs or algal meadows. For example, the yellow tailed emperor, *Lethrinus atkinsoni*, which is increasingly targeted by recreational fishers at Ningaloo [47], was only observed in algal meadows. Similarly, parrotfish (Family Scaridae) of the genus *Chlorurus* and *Scarus* are important herbivores and bioeroders [48], and these species were typically seen on coral reefs. Although common, these species were not always observed in their favored habitat, suggesting biological and physical attributes within coral and algal habitats may influence where juvenile fish occur. Alternatively this may reflect insufficient supply of recruits to saturate all available habitats, or differing levels of post-recruitment mortality in habitats.

Among the species found predominantly on coral reefs 14 were closely associated with live coral, including several species that do not feed or associate with live corals as adults. Previous studies have also found some fish species associate with fewer microhabitats as juveniles than as adults [28] and are more closely associated with live corals during the early stages of their benthic life history [49], [50], [51]. This may explain why fish species with no obvious dependence on coral as adults decline in abundance following extensive mortality [16].

Among the fish species that closely associate with live coral as juveniles, all but one were found in high abundance on corymbose corals. This may reflect optimal branch spacing within corymbose colonies, allowing movement of juveniles within the colony and refuge from predators. Conversely, branch spacing within plate corals may be too tight to permit easy movement by juvenile fish, whilst branching and foliaceous colonies may be too open, allowing access by predators. Similarly, massive and encrusting corals may not provide structural complexity at a spatial scale relevant to small bodied juvenile fish. Importantly corymbose corals are susceptible to mortality caused by thermal bleaching [52], [53], [54], disease [54] and outbreaks of coral predators [55], [56]. The incidence and intensity of these disturbances is expected to increase due to climate change or other anthropogenic activities [57], [58], [59], [60]. As a consequence, prevalence of corymbose corals may decline on reefs and the loss of this essential habitat could have a detrimental affect on the juvenile fish that rely on them for food and shelter.

The skeletons of dead corals may also act as important habitat for fish, providing refuge for a broad suite of species. Indeed, manipulative and natural experiments suggest the structural complexity provided by coral skeletons may be a more important determinant of fish diversity and abundance than live coral cover [22], [61], [62], [63]. Our study found that several juvenile species closely associated with coral skeletons, but did not closely associate with live coral. This included herbivouous species which, as adults, feed intensively on the epilithic algal matrix preventing reef overgrowth by macroalgae [24]. The recruitment and survival of these fish is therefore intrinsically linked to resilience and recovery of reefs following extensive coral mortality [64], [65]. Similarly, a collapse of coral skeletons and loss of structural complexity following widespread coral mortality can result in reduced abundance of juvenile fish, potentially constraining the size of future fisheries stocks [19]. Hence both live and dead corals provide essential habitat for juvenile fish. The loss of live corals and the structural complexity associated with their skeletons could have detrimental effects on recruitment, juvenile survival and, ultimately, the structure of adult fish communities.

The presence of ecologically and fisheries important species in either coral or macroalgal habitats, combined with inferences of ontogenetic shift by fish between these habitats, indicates that both habitats are essential and warrant protection. This lends support for the current emphasis on planning conservation using representative habitat areas of multiple habitat types within marine reserves. Furthermore, approximately a third of the species investigated here were closely affiliated with corals or their skeletons as juveniles. This is much higher than the 10% of fish that rely on live coral as adults, and emphasizes the importance of coral habitats during the early life history of reef fish. Of particular concern is the close association of many juvenile fish with corals susceptible to disturbances that are likely to intensify or become more frequent due to climate change and anthropogenic activities. Identifying and protecting reefs resilient to the effects of climate change should therefore be a conservation priority.

## References

[1] Doherty PJ, Sale PF (1991). Spatial and temporal patterns in recruitment.. The ecology of fishes on coral reefs.

[2] Jones GP, S PF (1991). Postrecruitment Processes in the Ecology of Coral Reef Fish Populations: A Multifactorial Perspective.. The ecology of fishes on coral reefs.

[3] Sale PF, Sale PF (1991). Reef fish communities: open nonequilibrial systems.. The ecology of fishes on coral reefs.

[4] Caselle JE, Warner RR (1996). Variability in recruitment of coral reef fishes: the importance of habitat at two spatial scales.. Ecology.

[5] Tolimieri N (1995). Effects of microhabitat characteristics on the settlement and recruitment of a coral reef fish at two spatial scales.. Oecologia.

[6] Shima JS (2001). Recruitment of a coral reef fish: roles of settlement, habitat, and postsettlement losses.. Ecology.

[7] Nagelkerken I, Roberts CM, van der Velde G, Dorenbosch M, Riel MC (2002). How important are mangroves and seagrass beds for coral-reef fish? The nursery hypothesis tested on an island scale.. Mar Ecol Prog Ser.

[8] Mumby PJ, Edwards AJ, Arias-Gonzalez JE, Lindeman KC, Blackwell PG (2004). Mangroves enhance the biomass of coral reef fish communities in the Caribbean.. Nature.

[9] Munday PL, Jones GP, Caley MJ (1997). Habitat specialisation and the distribution and abundance of coral-dwelling gobies.. Mar Ecol Prog Ser.

[10] Randall JE, Allen GR, Steene RC (1990). Fishes of the Great Barrier Reef and Coral Sea..

[11] Lieske E, Myers R (1996). Coral Reef Fishes..

[12] Adams AJ, Ebersole JB (2002). Use of back-reef and lagoon habitats by coral reef fishes.. Mar Ecol Prog Ser.

[13] Garpe KC, Öhman MC (2007). Non-random habitat use by coral reef fish recruits in Mafia Island Marine Park, Tanzania.. Afr J Mar Sci.

[14] Lecchini D, Galzin R (2005). Spatial repartition and ontogenetic shifts in habitat use by coral reef fishes (Moorea, French Polynesia).. Mar Biol.

[15] Chittaro PM, Fryer BJ, Sale PF (2004). Discrimination of French grunts (Haemulon flavolineatum, Desmarest, 1823) from mangrove and coral reef habitats using otolith microchemistry.. J Exp Mar Biol Ecol.

[16] Jones GP, McCormick MI, Srinivasan M, Eagle JV (2004). Coral decline threatens fish biodiversity in marine reserves.. Proc Natl Acad Sci USA.

[17] Pratchett MS, Munday PL, Wilson SK, Graham NAJ, Cinner JE (2008). Effects of climate-induced coral bleaching on coral-reef fishes: ecological and economic consequences.. Oceanogr Mar Biol.

[18] Wilson SK, Graham NAJ, Pratchett MS, Jones GP, Polunin NVC (2006). Multiple disturbances and the global degradation of coral reefs: are reef fishes at risk or resilient?. Global Change Biology.

[19] Graham NAJ, Wilson SK, Jennings S, Polunin NVC, Robinson J (2007). Lag effects in the impacts of mass coral bleaching on coral reef fish, fisheries, and ecosystems.. Cons Biol.

[20] Wilson SK, Fisher R, Pratchett M, Graham N, Dulvy N (2010). Habitat degradation and fishing effects on the size structure of coral reef fish communities.. Ecological Applications.

[21] Wilson SK, Dolman AM, Cheal AJ, Emslie MJ, Pratchett MS (2009). Maintenance of fish diversity on disturbed coral reefs.. Coral Reefs.

[22] Graham NAJ, Wilson SK, Jennings S, Polunin NVC, Bijoux JP (2006). Dynamic fragility of oceanic coral reef ecosystems.. Proc Natl Acad Sci USA.

[23] Gardner TA, Cote IM, Gill JA, Grant A, Watkinson AR (2003). Longterm region-wide declines in Caribbean corals.. Science.

[24] Bellwood DR, Hughes TP, Folke C, Nyström M (2004). Confronting the coral reef crisis.. Nature.

[25] Bruno JF, Selig ER (2007). Regional decline of coral cover in the Indo-Pacific: timing, extent, and subregional comparisons.. PLoS One.

[26] DeMartini EE, Anderson TW, Kenyon JC, Beets JP, Friedlander AM (2010). Management implications of juvenile reef fish habitat preferences and coral susceptibility to stressors.. Mar Freshw Res.

[27] Öhman MC, Munday PL, Jones GP, Caley MJ (1998). Settlement strategies and distribution patterns of coral-reef fishes.. J Exp Mar Biol Ecol.

[28] Wilson SK, Burgess S, Cheal A, Emslie M, Fisher R (2008). Habitat utilisation by coral reef fish: implications for specialists vs generalists in a changing environment.. Journal of Animal Ecology.

[29] Booth DJ, Beretta GA (2002). Changes in a fish assemblage after a coral bleaching event.. Mar Ecol Prog Ser.

[30] Pratchett MS, Marnane MJ, Berumen ML, Eagle JE, Pratchett DJ (2008). Habitat associations of juvenile versus adult butterflyfishes.. Coral Reefs.

[31] Wilson SK, Adjeroud M, Bellwood DR, Berumen ML, Booth D (2010). Crucial knowledge gaps in current understanding of climate change impacts on coral reef fishes.. Journal of Experimental Biology.

[32] Nagelkerken I, van der Velde G, Gorissen MW, Meijer GJ, van't Hof T (2000). Importance of mangroves, seagrass beds and the shallow coral reef as a nursery for important coral reef fishes, using a visual census technique.. Est Coast Shelf Sci.

[33] Unsworth KF, De León PS, Garrard SL, Jompa J, Smith DJ (2010). High connectivity of Indo-Pacific seagrass fish assemblages with mangrove and coral reef habitats.. Mar Ecol Prog Ser.

[34] Bancroft KP, Sheridan MW (2000). The major marine habitats of Ningaloo Marine Park and the proposed southern extension..

[35] Veron JE (2000). Corals of the World..

[36] Wilson SK, Graham NAJ, Polunin NVC (2007). Appraisal of visual assessments of habitat complexity and benthic composition on coral reefs.. Mar Biol.

[37] Sweatman HPA (1985). The influence of adults of some coral reef fishes on larval recruitment.. Ecol Monogr.

[38] Booth DJ (1992). Larval settlement patterns and preferences by domino damselfish Dascyllus albisella Gill.. J Exp Mar Biol Ecol.

[39] Wellington GM (1992). Habitat selection and juvenile persistence control the distribution of two closely related Caribbean damselfishes.. Oecologia.

[40] Depczynski M, Heyward A, Birrell C, Colquhoun J, Radford B (2010). Methods for monitoring the health of benthic communities. Perth, Australia: Field trip report for 2009. Western Australian Marine Science Institution (WAMSI).. Node 3 Project.

[41] Wade PR (2000). Bayesian methods in conservation biology.. Cons Biol.

[42] Ellison AM (2004). Bayesian inference in ecology.. Ecol Lett.

[43] Best NG, Cowles MK, Vines SK (1995). CODA: Convergence diagnosis and output analysis software for Gibbs sampling output, Version.

[44] Raftery A, Lewis S (1992). One long run with diagnostics: implementation strategies for Markov chain Monte Carlo.. Stat Sci.

[45] Ivlev VS (1961). Experimental ecology of the feeding of fishes..

[46] Manly BF, McDonald LL, Thomas DL (1993). Resource Selection by Animals..

[47] Sumner NR, Williamson PC, Malseed BE (2002). A 12-month survey of recreational fishing in the Gascoyne bioregion of Western Australia during 1998-99..

[48] Bellwood DR, Choat JH (1990). A functional analysis of grazing in parrotfishes (family Scaridae): the ecological implications.. Environ Biol Fish.

[49] Booth DJ, Beretta GA (1994). Seasonal recruitment, habitat associations and survival of pomacentrid reef fish in the US Virgin Islands.. Coral Reefs.

[50] Gutiérrez L (1998). Habitat selection by recruits establishes local patterns of adult distribution in two species of damselfishes: *Stegastes dorsopunicans* and *S. planifrons*.. Oecologia.

[51] Feary DA, Almany GR, McCormick MI, Jones GP (2007). Habitat choice, recruitment and the response of coral reef fishes to coral degradation.. Oecologia.

[52] Marshall PA, Baird AH (2000). Bleaching of corals on the Great Barrier Reef: differential susceptibilities among taxa.. Coral Reefs.

[53] Baird AH, Marshall PA (2002). Mortality, growth and reproduction in scleractinian corals following bleaching on the Great Barrier Reef.. Mar Ecol Prog Ser.

[54] Willis BL, Page CA, Dinsdale EA, Rosenberg E, Loya Y (2004). Coral disease on the Great Barrier Reef.. Coral health and disease.

[55] Pratchett MS (2007). Feeding preferences of *Acanthaster planci* (L.) under controlled conditions of food availability.. Pac Sci.

[56] Schoepf V, Herler J, Zuschin M (2010). Microhabitat use and prey selection of the coral-feeding snail Drupella cornus in the northern Red Sea.. Hydrobiologia.

[57] Harvell CD, Kim K, Burkholder JM, Colwell RR, Epstein PR (1999). Emerging marine diseases - Climate links and anthropogenic factors.. Science.

[58] Hoegh-Guldberg O (1999). Climate change, coral bleaching and the future of the world's coral reefs.. Mar Fresh Res.

[59] Dulvy NK, Freckleton RP, Polunin NVC (2004). Coral reef cascades and the indirect effects of predator removal by exploitation..

[60] Bruno JF, Selig EF, Casey KS, Page CA, Willis BL (2007). Thermal stress and coral cover as drivers of coral disease outbreaks.. PLoS Biology.

[61] Sano M, Shimizu M, Nose Y (1987). Long-term effects of destruction of hermatypic corals by Acanthaster planci infestation on reef fish communities at Iriomote Island, Japan.. Mar Ecol Prog Ser.

[62] Syms C, Jones GP (2000). Disturbance, habitat structure, and the dynamics of a coral-reef fish community.. Ecology.

[63] Garpe KC, Yahya SAS, Lindahl U, Ohman MC (2006). Effects of the 1998 coral bleaching event on reef fish assemblages.. Mar Ecol Prog Ser.

[64] Mumby PJ, Hastings A, Edwards HJ (2007). Thresholds and the resilience of Caribbean coral reefs.. Nature.

[65] Nyström M, Graham NAJ, Lokrantz J, Norstrom AV (2008). Capturing the cornerstones of coral reef resilience: linking theory to practice.. Coral Reefs.

