# Correction: Habitat Associations of Juvenile Fish at Ningaloo Reef, Western Australia: The Importance of Coral and Algae

**DOI:** 10.1371/annotation/53a56437-a810-4373-baee-16685ec20b2f

**Published:** 2011-01-07

**Authors:** Shaun K. Wilson, Martial Depczynski, Rebecca Fisher, Thomas H. Holmes, Rebecca A. O'Leary, Paul Tinkler

There was an error in reference #33. The correct reference is: Unsworth RKF, De León PS, Garrard SL, Jompa J, Smith DJ, et al. (2008) High connectivity of Indo-Pacific seagrass fish assemblages with mangrove and coral reef habitats. Mar Ecol Prog Ser 353: 213–224.

